# Supplementary figures and images for: Characterization of the bHLH family of transcriptional regulators in the acoel S. roscoffensis and their putative role in neurogenesis
Source: EvoDevo. 2018 Mar 29;9:8. doi: 10.1186/s13227-018-0097-y (PMC5875013; doi:10.1186/s13227-018-0097-y)

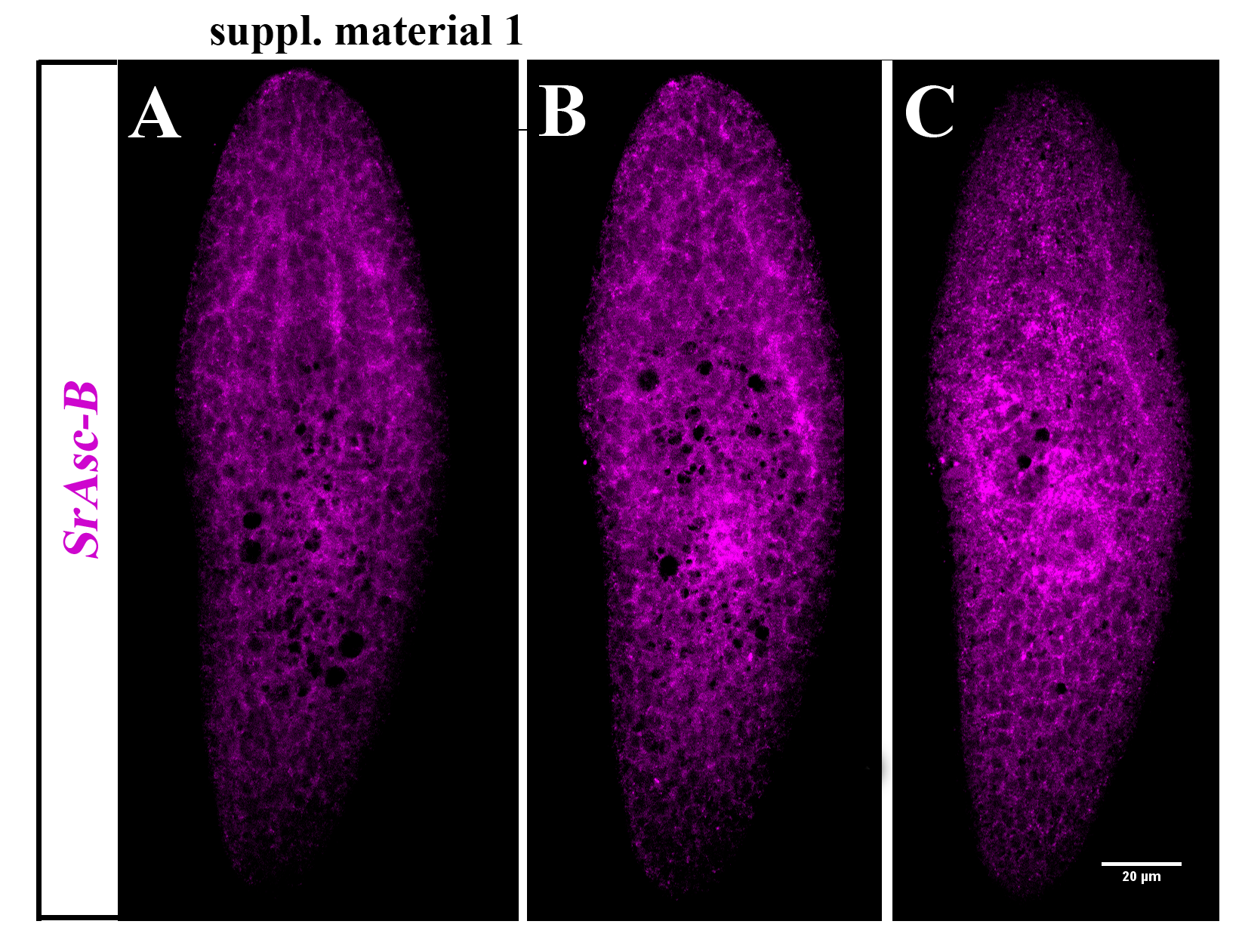

Supplement: Supplementary file 1 — Additional file 1: Fig. S1. Detailed expression of SrAscB (pink), Achaete-Scute gene family ortholog B in (aprox.) 24 h juvenile of the acoel S. roscoffensis. Panels correspond to three different planes along the dorso-ventral axis. Scale bar 20 µm. [file 13227_2018_97_MOESM1_ESM.tiff]

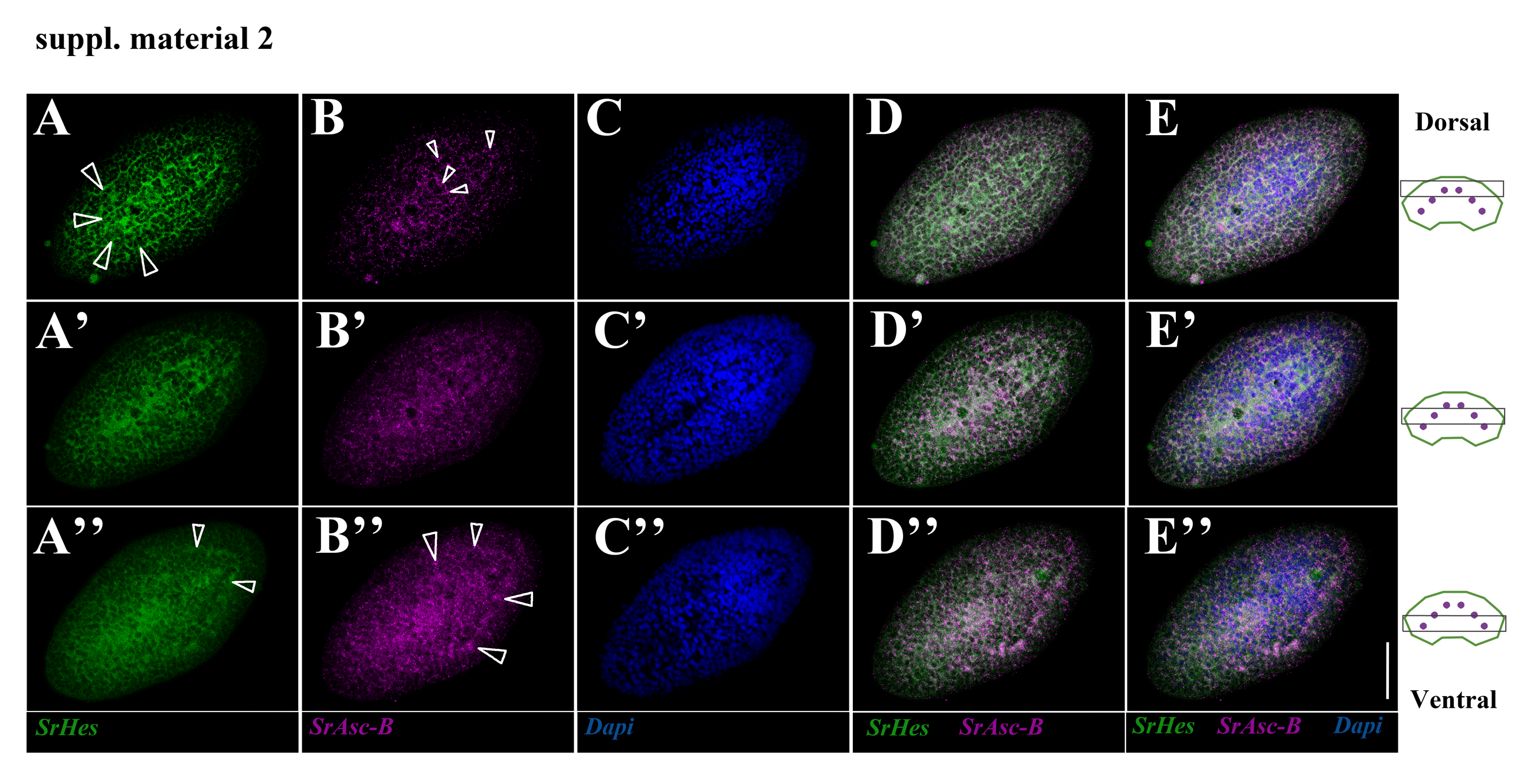

Supplement: Supplementary file 2 — Additional file 2: Fig. S2. Detailed expression domains of SrAscB (pink) and SrHes/Hey (green) genes in, approx., 24 h juveniles of the acoel S. roscoffensis. The different rows correspond to their different planes along the dorso-ventral axis of the juvenile. In the bottom line of every column, it is indicated the gene combination used, except in C, C′ and C′′, which correspond to Dapi stainings. Arrowheads point to the expression domain within the nervous system. Scale bar 40 µm. [file 13227_2018_97_MOESM2_ESM.tiff]
